# Mobilized Multipotent Hematopoietic Progenitors Promote Expansion and Survival of Allogeneic Tregs and Protect Against Graft Versus Host Disease

**DOI:** 10.3389/fimmu.2020.607180

**Published:** 2021-02-12

**Authors:** Maud D’Aveni, Anne-Béatrice Notarantonio, Viviane A. Agbogan, Allan Bertrand, Guillemette Fouquet, Pauline Gastineau, Meriem Garfa-Traoré, Marcelo De Carvalho, Olivier Hermine, Marie-Thérèse Rubio, Flora Zavala

**Affiliations:** ^1^ Université de Lorraine, CHRU Nancy, Hematology Department, Nancy, France; ^2^ Université de Lorraine, UMR 7365 CNRS, IMoPA, Nancy, France; ^3^ Department of Immunology, Infectiology and Haematology, Université de Paris, Inserm U1151, CNRS UMR 8253, Institut Necker Enfants Malades (INEM), Paris, France; ^4^ Université de Paris, INSERM UMR 1163, Imagine Institute, Laboratory of Cellular and Molecular Mechanisms of Hematological Disorders and Therapeutic Implications, Paris, France; ^5^ Université de Paris, SFR Necker-UMS 3633/US24-Structure Fédérative de Recherche Necker, Plateforme d’Imagerie Cellulaire, Paris, France; ^6^ Université de Lorraine, CHRU Nancy, Immunology Department, Nancy, France

**Keywords:** allogeneic HSCT, graft versus host disease, mobilization, multipotent progenitors, regulatory T cells, expansion, alloreactivity, mixed lymphocyte reaction

## Abstract

Allogeneic Hematopoietic Stem Cell Transplantation (Allo-HSCT) is routinely performed with peripheral blood stem cells (PBSCs) mobilized by injection of G-CSF, a growth factor which not only modulates normal hematopoiesis but also induces diverse immature regulatory cells. Based on our previous evidence that G-CSF-mobilized multipotent hematopoietic progenitors (MPP) can increase survival and proliferation of natural regulatory T cells (Tregs) in autoimmune disorders, we addressed the question how these cells come into play in mice and humans in an alloimmune setting. Using a C57BL/6 mouse model, we demonstrate that mobilized MPP enhance the immunosuppressant effect exerted by Tregs, against alloreactive T lymphocytes, both *in vitro* and *in vivo.* They do so by migrating to sites of allopriming, interacting with donor Tregs and increasing their numbers, thus reducing the lethality of graft-versus-host disease (GVHD). Protection correlates likewise with increased allospecific Treg counts. Furthermore, we provide evidence for a phenotypically similar MPP population in humans, where it shares the capacity to promote selective Treg expansion *in vitro.* We postulate that G-CSF-mobilized MPPs might become a valuable cellular therapy to expand donor Tregs *in vivo* and prevent GVHD, thereby making allo-HSCT safer for the treatment of leukemia patients.

## Introduction

Allogeneic Hematopoietic Stem Cell Transplantation (allo-HSCT) is the only therapy that can cure some hematological malignancies resistant to standard anti-cancer treatment. Its success relies on the capacity of donor T cells to eliminate residual tumor cells, called the Graft-versus-Tumor or GVT effect. The drawback is that donor T cells also target alloantigens expressed on the cells of the recipient’s organs, which leads to life-threatening complications, resulting in graft-versus-host disease (GVHD). Administration of Granulocyte Colony Stimulating Factor (G-CSF) is routinely used for allo-HSCT as a procedure to mobilize and harvest peripheral blood stem cells (PBSC) from healthy donors. Even though PBSC comprise a higher proportion of T cells than bone marrow (BM) allografts, they do not increase the incidence of acute graft-versus-host disease (GVHD) (1). To explain this paradox, we and other investigators have demonstrated that mobilization with G-CSF induces myeloid-derived suppressor cells (MDSCs) capable of reducing alloreactive T lymphocyte proliferation (2). Among these, some subsets have been associated with a lower incidence of acute GVHD. Unfortunately, in the context of alloimmunity, suppressing conventional T cells could enhance infection and relapse incidence (3). This adverse effect led to the implementation of an alternative strategy to enhance the contribution of regulatory T cells (Tregs). It has indeed been reported that freshly isolated donor Tregs (4, 5) or *ex vivo* expanded Tregs (6) added to donor CD3^+^ T cells early after allo-HSCT, reduced GVHD, while maintaining the GVT effect. We knew also that G-CSF could mobilize murine CD117^+^Sca-1^+^CD34^+^CD11b^−/low^ multipotent progenitor precursor cells (mobMPP), which promoted, in turn, the expansion of regulatory T cells. The latter prevented spontaneous autoimmune type 1 diabetes in the NOD mouse model (7, 8) and modulated experimental autoimmune encephalomyelitis (EAE) in the C57BL6/J mouse model (9). These findings prompted us to investigate whether mobMPP could likewise contribute to Treg expansion during allo-HSCT. Having confirmed that *in vitro* these progenitors did effectively enhance Treg survival and proliferation in both mice and humans, we examined whether they could protect recipient mice from acute GVHD and improve their survival through expansion of natural allo-antigen-specific donor Tregs early after allo-HSCT. Furthermore, we verified whether human G-CSF-mobilized MPP shared similar properties.

## Materials and Methods

### Mice

BALB/cJ (H2^d^), C3H/HeJ (H2^d^) and C57BL6/J (H2^b^) mice were purchased from Janvier (Le Genest Saint Isle, France). Congenic (CD45.1) C57BL6/J (H2^b^), Foxp3^GFP^-C57BL6/J and CD45.1-Foxp3^GFP^-C57BL6/J mice were kindly provided by Lucienne Chatenoud (Institut Necker Enfants Malades, INSERM U1151, Paris, France). All mice were bred and housed in a specific pathogen-free facility in microisolator cages and used at 8–12 weeks of age in protocols approved by the local Ethical Committee (CEEA34.0AP.018.11).

### Recovery of mobMPP cells

C57BL6/J mice (B6) were injected subcutaneously for 5 consecutive days with recombinant human G-CSF (200 μg/kg/day) (Neupogen, Amgen, France) and recombinant murine Flt3-L (20 μg/kg/day) (Immunotools, Friesoythe, Germany). At day 6, spleen cells labeled with mAbs directed against mature lineage markers (CD4, CD8, Gr-1, CD45R, Ter119, CD11b) were depleted with anti-rat Ig-coated magnetic beads (Invitrogen Dynal, ThermoFisher Scientific, Cergy Pontoise, France). The lineage negative (lin^−^) fraction was electronically sorted, as previously described (7). Briefly, mobMPP were stained with appropriately labeled mAbs: CD34, CD117, Sca-1, CD11b (eBioscience), after incubation with purified anti-CD16/32-mAbs to block non-specific FcR binding and electronically sorted as CD117^+^Sca-1^+^CD34^+^CD11b^−/low^ cells.

Human peripheral blood samples (10 ml EDTA-tube) from rhG-CSF-treated (filgastrim 10 µg/kg/day for 5 consecutive days) healthy allogeneic donors (randomly selected at Necker Hospital and Saint Antoine Hospital in Paris) were collected after informed consent. Ficoll-purified peripheral PBMC were stained with the following antibodies: CD34, CD38, CD45RA, CD90 (eBioscience, Life Technologies, Villebon-sur-Yvette, France) and sorted (FACS Aria II, BD Bioscience, Le Pont de Claix, France) as CD34^+^CD38^+^CD90^−^CD45RA^−^ mobilized MPP cells, and mobilized hematopoietic stem cells (mobHSC) identified by their CD34^+^CD38^−^ CD90^+^CD45RA^−^ phenotype.

### Proliferation Assays

In mice, conventional T cells from B6 mice were purified by negative selection using a CD4 T cell depletion kit (Miltenyi Biotec, Bergisch-Gladbach, Germany). Purity was routinely above 98%. Natural Tregs were stained with appropriately labeled mAbs against CD4 and CD25 and electronically sorted as CD4^+^CD25^high^.

T cell activation (including conventional CD4^+^ T cells, conventional CD4^+^CD25^−^ T cells and natural CD4^+^CD25^high^ Tregs) was performed with allogeneic dendritic cells or, alternatively by non-specific TCR activation, with anti-CD3 and CD28 antibodies. Mature dendritic cells were harvested from the spleen of BALB/cJ (or C3H/HeJ) mice with a pan-DC selection kit (Miltenyi Biotec). B6 T cells were stained with 5 μM carboxyfluorescein succinimidyl ester (CFSE) (Invitrogen, Life Technologies, Villebon-sur-Yvette, France). In Mixed Lymphocyte Reaction (MLR), B6 T cells were mixed with DC stimulators at a ratio of five T cells for one DC, and plated in a 96-well round bottom culture plate (5.10^4^ T cells/well). In some experiments, mobMPP were added at 5.10^4^/well (1:1 T cell ratio), 10.10^4^/well (2:1 T cell ratio) or 15.10^4^/well (3:1 T cell ratio). In some experiments, Tregs were added at 5.10^4^/well (1:1 T cell ratio) alone or with mobMPP at a 1:1:1 T cell ratio. Non-specific Treg activation was performed in a 96 round bottom plate coated with 10 µg/ml anti-CD3 mAb (clone 2C11) and 10 µg/ml anti-CD28 mAb (Biolegend) for 2 h before the addition of T cells (50,000/well). In some experiments, monoclonal IL-2 blocking antibody was used at 10 µg/ml (R&D Systems). Cells were incubated in custom RPMI 1640 medium supplemented with 10% fetal calf serum, 50 µM 2-mercaptoethanol and 100 U/ml penicillin-streptomycin (Life Technologies, Villebon-sur-Yvette, France).

Conventional human T cells were purified by negative selection using a pan T cell depletion kit (Miltenyi Biotec). Purity was routinely above 98%. Human naïve Tregs were sorted (FACS Aria II, BD Bioscience, le Pont de Claix, France) as CD3^+^CD4^+^CD25^+^CD45RA^+^ cells and conventional T cells were sorted ad CD3^+^CD4^+^CD25^−^ T. They were plated at 50,000 cells/well in round-bottom 96-well microculture plates with or without mobMPP at a 1:1 T cell ratio in a final volume of 200 μl. Non-specific T cell activation was induced with 10 µg/ml anti-CD3 mAb (clone UCHT1, R&D Systems) and 10 µg/ml anti-CD28 mAb (clone 37407, R&D systems) for 2 h before adding T cells (50,000/well). In some experiments, monoclonal IL-1β blocking antibody was used (mouse anti-human clone 2805, R&D Systems).

### Flow Cytometry

*In vivo*, for some experiments, allo-HSCT was performed with T cells from congenic CD45.1-Foxp3^GFP^ B6 mice. Thereafter, donor natural Tregs were analyzed at days 8 and 30 post-transplantation, in lymph nodes and spleen using mAbs against CD45.1, CD3, CD4, CD25 (eBioscience).

*In vitro*, cultured Tregs were characterized with anti-CD4 and anti-CD25 (eBioscience). Foxp3 was measured following surface staining with fixation/permeabilization working solution according to the manufacturer’s guidelines (eBioscience). Intracellular IL-2 cytokine (eBioscience) staining was performed after a 3-h incubation of cultured Tregs +/− mobMPP with PMA (10 ng/ml) plus ionomycin (500 ng/ml) and Brefeldin A (2 mg/ml) at 37°C and 5% CO_2_.

In human healthy donors, mobMPP were stained with appropriately labeled mAbs against CD34, CD38, CD45RA, CD90, CD13 and CD33 (eBioscience). Tregs were labeled with anti- CD3, -CD4, -CD25 and -CD45RA, (eBioscience). To confirm that naïve Tregs were all Foxp3^+^, sorted naïve Tregs CD3^+^CD4^+^CD25^+^CD45RA^+^ were fixed/permeabilized (Fix/Perm solution, eBioscience) and stained according to the manufacturer’s instructions (eBioscience). For flow cytometric characterization of freshly sorted mobMPP, intra-cellular cytokine (IL-1β) staining was performed after 3-h incubation with brefeldin A (2 mg/ml). Flow cytometric characterization of Tregs +/−mobMPP after 4 days of culture, intracellular cytokine (IL-2, IFN-γ and TNF-α, eBioscience) staining was performed after 3-h incubation with PMA (10 ng/ml) and ionomycin (500 ng/ml) and brefeldin A (2 mg/ml) at 37°C and 5% CO_2_. Samples were analyzed on a FACS Canto II cytometer (BD Biosciences) using FlowJo software (Treestar, Ashland, OR).

### Acute GVHD

BALB/cJ recipients were lethally irradiated using 600 cGy total body X-ray irradiation on day -1 followed by intravenous caudal vein infusion of 10^7^ T cell-depleted B6 donor bone marrow (BM) cells, 2 × 10^6^ purified B6 T cells, with or without 0.35 × 10^6^ B6 mobMPP on day 0. Control groups were transplanted with BM cells alone (syngenic group) or BM + T cells (control T cell group). For some experiments, 2 × 10^6^ purified CD45.1-Foxp3^GFP^-B6 T cells were used for allo-HSCT to analyze donor Tregs on day-8 and day-30 post-HSCT by flow cytometry. In other experiments, allo-HSCT was carried out with 0.35 × 10^6^ CD45.1B6 mobMPP and 2 × 10^6^ purified CD45.2-Foxp3^GFP^-B6 T cells to evaluate donor mobMPP in lymph nodes and spleen at day-15 post-HSCT by confocal microscopy. AlloHSCT was performed with donor cells lacking Tregs, using BALB/cJ recipients having received a lethal 600 cGy total X-ray body irradiation on day- -1 followed by intravenous injection into the caudal vein of 10^7^ T cell-depleted B6 donor bone marrow (BM) cells, 2 × 10^6^ purified B6 CD25^−^ T cells (control CD25^−^ T cells group), and +/− 0.35 × 10^6^ B6 mobMPP on day-0. All recipient mice were monitored daily for survival, and at least twice a week for weight and acute GVHD score. The clinical scoring system was based on six parameters as previously described (2): weight loss, posture, activity, fur texture, skin integrity, and diarrhea. A severity scale of 0–2 was used for each parameter, with a maximum global score of 12.

### Confocal Microscopy Analysis of Recipient Lymph Nodes and Spleens

Spleens and lymph nodes from allografted BALB/c j mice were harvested on day 15 and immediately embedded in OCT (Sakura), snap frozen in liquid nitrogen, and stored at -80°C. Cryosections (7–9 μm) were cut, fixed in cold (-20°C) acetone for 10 min, rehydrated in washing buffer (TBS, pH 7.6), and exposed to blocking buffer (0.5% BSA and 10% goat serum in PBS) for 30 min at room temperature. Sections were then treated for 60 min at room temperature with rat anti-mouse CD3 and mouse biotin anti-CD45.1 antibody (eBioscience). Controls were set up without primary antibodies. Sections were washed 3–4 times, incubated with the secondary antibody followed by conjugated streptavidin-CY5 (20 min at room temperature). Sections were washed and mounted in Fluoromont-G (Southern Biotech). Images were acquired by confocal microscopy with a LSM 700 (Zeiss). Fluorescence of single channels was measured, and control reference was systematically done. Pictures were taken at 40× magnification. Images were analyzed and processed with ImageJ version 1.46.

### Statistical Analysis

The results were averaged in each group. Statistical analyses were performed using GraphPad Prism software version 8, and data are presented as mean ± SEM. Before analysis, the normality assumption was examined. Comparisons were performed by one-way ANOVA with Tukey’s multiple comparison when comparing more than two groups with normal distribution of values, by unpaired *t*-test for comparing two groups with normal distribution of values. Survival curves were compared by log-rank test. A *p*-value < 0.05 was considered statistically significant.

## Results

### MobMPP Inhibit Alloreactive T Cell Proliferation by Expanding Alloreactive Tregs in Mice

**Figure 1:**
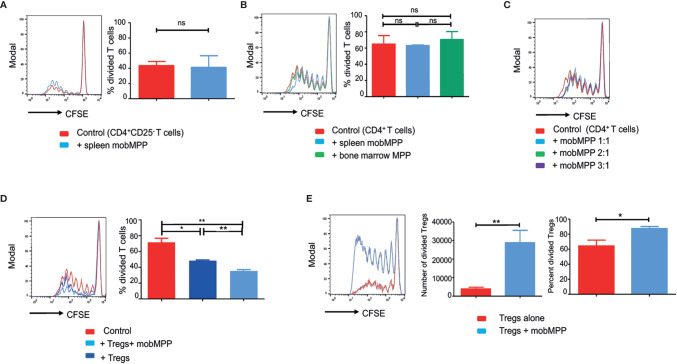
G-CSF-mobilized MPP can suppress MLR by enhancing Foxp3^+^ Treg proliferation. **(A)** CFSE dilution was assessed in B6 CD4^+^CD25^−^ T cell (50,000/well) activated by BALB/c DC (10,000/well) cultured alone (red) or with splenic mobMPP (blue) at a 1:1 ratio (50,000 CD4^+^CD25^−^ T:50,000 mobMPP/well). Percentage of divided T cells were 43.5± 3.17% in control, versus 41.23± 8.9% in coculture with mobMPP (*p* = 0.80). Data from 3 independent experiments (n = 3) were compared using Student’s unpaired *t*-test (ns, not significant). **(B)** Total B6 CD4^+^ T cells (containing both CD25^−^ and CD25^+^ CD4^+^ T-cells) activated by BALB/c dendritic cells were cultured alone (red), with splenic mobilized MPP at a 1:1 ratio (light blue), or with non-mobilized bone marrow MPP at a 1:1 ratio (green). Percentages of divided T cells were 64.88± 10.48% in control, versus 63 ± 1.30% and 70.43 ± 17.19% in coculture with mobMPP (*p* = 0.87) or bone marrow MPP (*p* = 0.73), respectively. Data were compared using one-way ANOVA with Tukey’s multiple comparison test (ns, not significant). **(C)** Total B6 CD4^+^ T cells (containing both CD25^−^ and CD25^+^ CD4^+^ T-cells) CFSE dilution is similar when B6 CD4^+^ T cells activated by BALB/c DC were cultured alone or with mobMPP at 1:1 ratio (50,000 CD4^+^ T cells:50,000 mobMPP/well). A higher number of MobMPP in the well (1:2 ratio (50,000 CD4^+^ T cells: 100,000 mobMPP/well), or 1:3 ratio (50,000 CD4^+^ T cells: 150,000 mobMPP/well) does not impact total B6 CD4^+^T cell proliferation (one experiment, n = 1). **(D)** B6 CD4^+^CD25^−^CD45.1 T cells activated by BALB/c DC were cultured alone (red) or with CD45.2 Tregs at a 1:1 ratio (dark blue), or with CD45.2 Tregs and CD45.2 mob-MPP at a 1:1:1 ratio (light blue) (n = 3). Percentages of CD45.1 divided T cells were respectively 71.54 ± 5.27% in control, 48.52 ± 0.86% in the presence of Tregs, (*p* = 0.0125, *) and 35.49 ± 1.66% with both Tregs and mobMPP, (**p = 0.0028). The percentage of divided T cells was higher when both Tregs and mobMPP were present, rather than Tregs alone (**p = 0.0023). Data were compared using Student’s unpaired *t*-test (ns, not significant). **(E)** B6 Treg cells activated with allogeneic BALB/c DC were cultured alone (red) or with mobMPP (blue) (n = 11). The number of Tregs in proliferation (≥ division 1) is lower when Tregs are cultured alone (3918 ± 905 vs. 28792 ± 6659, **p = 0.0014). Percentages of Tregs in proliferation (≥ division 1) are also lower when Tregs are cultured alone (64.28 ± 25.66% vs. 87.40 ± 9.26%, *p = 0.0108, using Student’s unpaired *t*-test).

We evaluated the effect of splenic mobMPP on alloreactive T cell proliferation induced by allogeneic dendritic cells during mixed lymphocyte reaction (MLR). We failed to observe a significant immunosuppression when either CD4^+^CD25^−^ T cells (**Figure 1A**) or total CD4^+^ (including CD25^+^ and CD25^−^) T cells (**Figure 1B**) were used, even if mobMPP were added at higher ratios (**Figure 1C**). By contrast, mobMPP became strongly immunosuppressant on alloreactive T cell proliferation when cultured with Tregs at a 1:1:1 ratio (**Figure 1D**). In fact, we observed that Tregs activated by allogeneic dendritic cells or alternatively by anti-CD3 and -CD28 antibodies, were expanded in terms of cell counts and proportion of divided cells, assessed by CFSE dilution, from day-4 of co-cultures (**Figure 1E**). To ensure that expanding Treg cells maintain their phenotype, we studied activated Tregs (+/− mobMPP) after 4 days of culture. We observed that Tregs maintained their CD4^+^CD25^high^Foxp3^+^ phenotype (**Supplementary Data, Figure S1A**) and did not produce IL-2 in contrast with activated conventional T cells (**Supplementary Data, Figure S1B**). Notably, we observed that mobMPP did not produce IL-2 either (**Figure S1B**). In addition, Treg expansion by mobMPP occurred even in the presence of neutralizing anti-IL-2 antibody, demonstrating that mobMPP promoted Treg expansion by an IL-2 independent mechanism (**Supplementary Data, Figure S1C**).

### Adoptively Transferred mobMPP Prevent Acute GVHD and Improve Donor Treg Expansion

**Figure 2:**
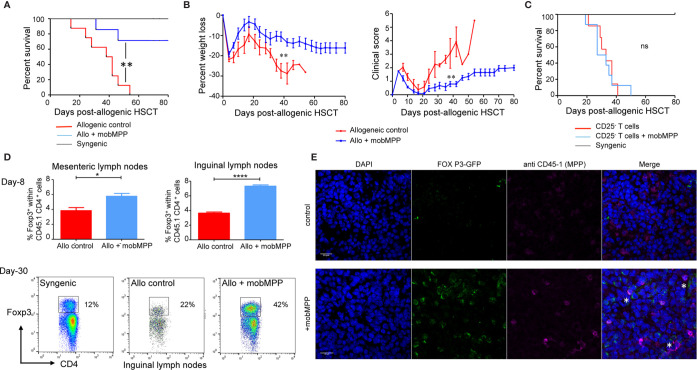
G-CSF mobilized MPP prevent GVHD by enhancing Foxp3^+^ Tregs *in vivo*. **(A)** Lethally irradiated (6 Gy) BALB/cJ recipients received 1 × 10^7^ B6 T cell-depleted BM cells alone (black, n = 4) or together with 2 × 10^6^ purified B6 T cells (allogeneic control, red, n = 7). In a third group 0.35 × 10^6^ B6-derived mobMPP were co-transferred with the two other populations (blue, n = 7) on day 0. Less than 40% of mice having received co-transferred mobMPP died over 80 days whereas median survival was 40 days without mobMPP (**p = 0.004). Data shown represent pooled results from 2 independent experiments. Results were compared with Kaplan-Meier survival curves. **(B)** Mean weight loss of BALB/cJ recipients at day 40 post-HSCT (median survival group) was: -10.57 ± 2.28% in mice co-injected with mobMPP (n = 6) vs. -27.44% ± 3.60% in allogeneic controls (n = 4). Mean clinical GVHD scores in recipients at day 40 post-HSCT was: 0.8 ± 0.19 in mice co-injected with mobMPP (n = 6) vs. 3.06 ± 1.1 in allogeneic controls (n = 4). Data were compared using Student’s unpaired *t*-test (**p ≤ 0.01). **(C)** Lethally irradiated (6 Gy) BALB/cJ recipients received 1 × 10^7^ B6 T-cell depleted BM cells alone (black, n = 4), with 2 × 10^6^ purified CD25^−^ B6 T cells (allogeneic control, red, n = 7) or with additional 2 × 10^6^ purified CD25^−^ B6 T cells and 0.35 × 10^6^ B6-derived mobMPP (blue, n = 7). Median survival in mice having received allogeneic HSCT plus CD25^−^ T cells was 30 days vs. 33 days in mice with co-transferred mobMPP (*p* = 0.52). Results were compared with Kaplan-Meier survival curves (ns, not significant). **(D)** Percentage of Foxp3^+^ Tregs in allogeneic CD45.1^+^CD4^+^ B6 T cells were studied in control BALB/c mice (red) or in BALB/c mice transplanted with mobMPP (blue) (n = 3). The percentage of Tregs in the CD45.1^+^CD4^+^ T cells in mesenteric lymph nodes were 3.84 ± 0.71% vs. 5.76 ± 0.67%, (*p=0.0274), respectively. The percentage of Tregs in the CD45.1^+^CD4^+^ T cells in inguinal lymph nodes were 3.64 ± 0.28 vs. 7.31 ± 0.29%, (****p < 0.0001, using Student’s unpaired t-test). **(E)** Representative confocal fluorescence micrograph of inguinal lymph nodes. BALB/c mice were sacrificed at day 15 post-allogeneic HSC and inguinal lymph nodes were harvested for confocal microscopy. Top: inguinal lymph node from control allografted BALB/c mice. Below: inguinal lymph node from allografted BALB/c mice with CD45.1 mobMPP (DAPI, blue; Foxp3, green; CD45.1 red; merged images show Foxp3/CD45.1 colocalization, indicated by *). Bar: 0.2 μm.

We further investigated how mobMPP affected allogeneic HSCT in a murine model. Donor cells were electronically sorted as lin^−^, CD117^+^, Sca-1^+^ CD34^+^, CD11b^−^ cells from the spleen of B6 mice having received G-CSF and Flt3-L injections (mobMPP). BALB/c mice allografted with 2.10^6^ T cells together with mobMPP showed a significantly improved survival, as compared to allogeneic controls without mobMPP (**Figure 2A**). This group of recipients was protected against GVHD and weight loss (**Figure 2B**). Mice allografted with 2.10^6^ CD25^−^ T cells (devoid of Tregs) together with mobMPP did not survive significantly longer than allogeneic controls without mobMPP (**Figure 2C**), underlining the strict requirement for Tregs for the protective effect of mobMPP. Eight days after allo-HSCT, we found that mesenteric and inguinal lymph nodes contained an increased percentage of Tregs among the donor CD45.1 CD4^+^ population, by contrast with spleen. This percentage could reach 42% one month after allo-HSCT in surviving recipients (**Figure 2D**). This accumulation of Tregs in recipient inguinal lymph nodes was confirmed by confocal microscopy, which revealed also that mobilized MPP and expanded Tregs were in close contact with each other (**Figure 2E**).

### Adoptive Transfer of mobMPP Modulates Alloreactive T Cell Proliferation In Vivo

**Figure 3:**
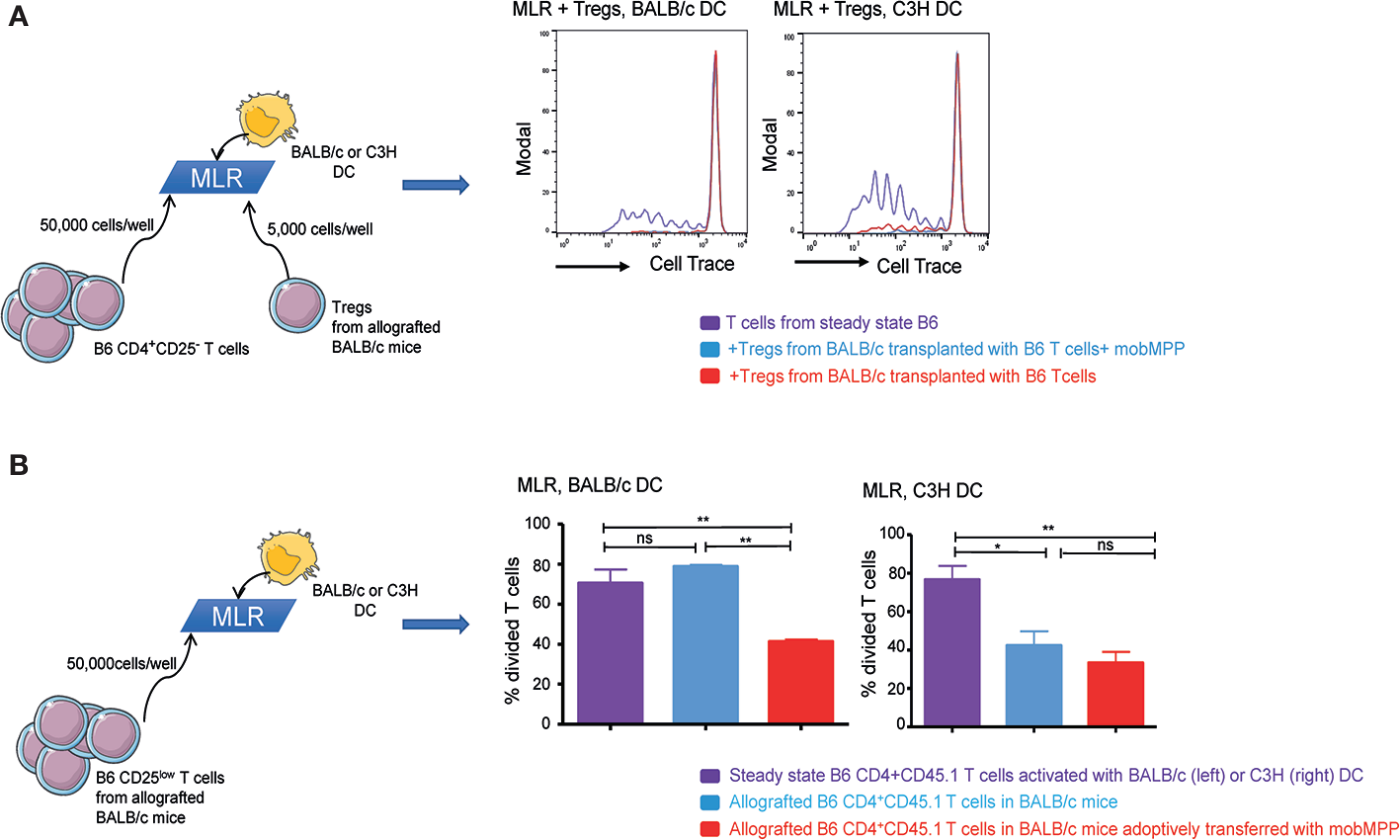
G-CSF mobilized MPP induce tolerance. **(A)** Steady state B6 CD4^+^CD45.1^+^ T cells activated by BALB/c (left) or C3H (right) DC were cultured alone (purple) or with Tregs in 10:1 ratio (50,000 T cells: 5,000 Tregs). Tregs were sorted from the spleen of allografted control BALB/c mice (red), or from allografted BALB/c mice with mobMPP (light blue) (n = 3). CFSE dilution within CD45.1^+^ T cells were compared. (One representative experiment out of 3). **(B)** Left: Steady state B6 CD4^+^CD25^low^CD45.1^+^ T cells activated by BALB/c DC were analyzed for CFSE dilution (purple) and compared to CFSE dilution of allografted B6 CD4^+^CD25^low^CD45.1^+^ T cells in BALB/c mice with (light blue) or without (red) adoptive transfer of mobMPP. Percentage of steady state B6 CD4^+^CD25^low^CD45.1^+^ T cells in division is 70.57 ± 6.81% (purple) vs. 41.37 ± 1.03% (red), vs. 70.57 ± 6.81% (light blue), respectively **p ≤ 0.005 by one-way ANOVA with Tukey’s multiple comparisons test. Right: Steady state B6 CD4^+^CD25^low^CD45.1^+^ T cells activated by C3H dendritic cells were analyzed for CFSE dilution (purple) and compared to CFSE dilution of allografted B6 CD4^+^CD25^low^CD45.1^+^ T cells in C3H mice with (light blue) or without (red) adoptive transfer of mobMPP. Percentage of steady state B6 CD4^+^CD45.1 T cells in division is 76.60 ± 7.18% (purple) vs. 42.40 ± 7.35% (red), vs. 33.37. ± 5.69% (light blue), respectively, n = 3, (*p = 0.0275, **p = 0.0097; ns, not significant, by one-way ANOVA with Tukey’s multiple comparisons test).

We further assessed whether *in vivo* expanded Tregs exhibited specific alloreactive immunosuppressive functions. For this purpose, we performed MLR with 50,000 CD25^−^ B6 T cells/well. At day-8 post-transplantation, Tregs expanded from B6 CD45.1 donor cells in BALB/c recipients in the presence or absence of mobMPP were electronically sorted and added during MLR, at a very low ratio (10 T cell: 1 Treg). We observed that *in vivo* expanded Tregs could totally suppress MLR when B6 T cells were activated not only by BALB/c but also by C3H DC, demonstrating that *in vivo* expanded Tregs were highly suppressive and capable of hampering alloreactive responses non-specifically (**Figure 3A**). We further tested CD45.1^+^CD4^+^CD25^low^ allografted T cells, on day 8 after transplantation. During MLR, only CD4^+^CD25^low^ T cells sorted from BALB/c mice transplanted with mobMPP exhibited a significantly reduced proliferation after stimulation by BALB/c DC, compared with proliferation of CD4^+^CD25^low^ T cells from allografted mice that had not received mobMPP. However, this suppression did not occur in the presence of C3H DC, suggesting that natural Tregs already suppressed alloreactive CD25^low^ T cells from day-8 post-transplantation onwards (**Figure 3B**). Therefore, mobilized MPP have contributed to expand highly suppressive donor natural Tregs that immediately suppressed alloreactive T cells in BALB/c recipients. It can therefore be concluded that *in vivo* Treg expansion by mobMPP confers allo-antigen specific T cell suppression to control GVHD in allo-HSCT.

### Characterization of a Human mobMPP Counterpart Mobilized by G-CSF

**Figure 4:**
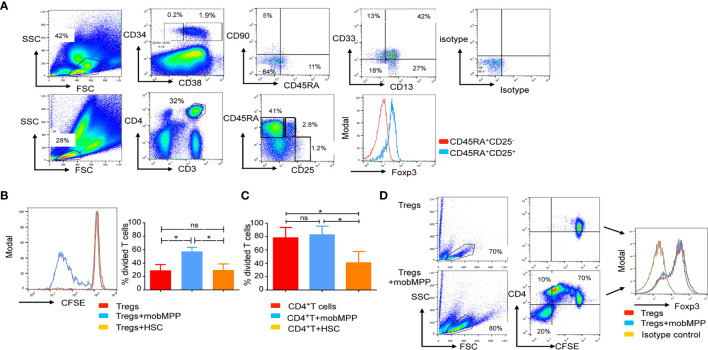
Human mobilized MPP share features of immature myeloid cells endowed with naïve Treg proliferation properties. **(A)** Human G-CSF mobilized peripheral blood stem cells (PBSC) were analyzed by flow cytometry. We first distinguished the CD34^+^ fraction from the CD34^−^ fraction of cells. In the gated CD38^+^ CD34^+^ fraction, a population of cells that lacked CD90 and CD45RA, homogeneously expressed CD13 and CD33 and was identified and termed mobMPP. Mobilized hematopoietic stem cells (mobHSC) were distinguished as CD34^+^CD38^−^CD45RA^−^CD90^−^. Below, in the same PBSC, naïve Tregs defined as CD3^+^CD4^+^CD45RA^+^CD25^+^ were analyzed by flow cytometry. All gates were based on isotype control. **(B)** Anti-CD3 and -CD28 activated naïve Treg cell proliferation after 4 days of culture alone (red) with mobMPP (light blue) or with mobHSC (orange) at a 1:1 ratio (n = 3). Percentages of divided naïve Tregs were in control = 29.17 ± 8.58% vs. 60.77 ± 2.847% with mobMPP vs. 29.67 ± 9.06% with mobHSC, respectively. Data were compared using one-way ANOVA with Tukey’s multiple comparison test (ns, not significant, *p = 0.0371). **(C)** Anti-CD3 and -CD28 activated CD4^+^CD25^−^ T cell proliferation after 4 days of culture alone (red) with mobMPP (light blue) or with mobHSC (orange) at a 1:1 ratio (n = 3). Percentages of divided T cells were in control = 78.03 ± 8.945 vs. 82.57 ± 7.583% with mobMPP, vs. 40.70 ± 9.815% with mobHSC respectively. Data were compared using one-way ANOVA with Tukey’s multiple comparison test (ns, not significant, *p =0.0289). **(D)** Naïve Treg cells activated by anti-CD3 and anti-CD28 were cultured alone or with mobMPP. After 4 days of culture, intra-nuclear Foxp3 was measured.

We analyzed the CD34^+^ fraction of PBSC from 30 healthy donors. It has been previously reported that human HSC are enriched in the Lin^−^CD34^+^CD38^−^CD90^+^ cell compartment of non-mobilized cord blood and bone marrow cells. By contrast, in mobilized PBSCs, the majority of the CD34^+^ cells are CD38^+^CD90^−^CD45RA^−^ and homogeneously expressed low levels of CD13 and CD33 (**Figure 4A**). We distinguished mobMPP as CD34^+^CD38^−^CD90^−^CD45RA^−^ and mobilized hematopoietic stem cells (mobHSC) as CD34^+^CD38^−^CD90^+^CD45RA^−^. We tested these two main subsets in co-culture with naïve Tregs (described in **Figure 4A**). Only the CD34^+^CD38^+^CD90^−^CD45RA^−^ subset (mobMPP) could increase naïve Treg proliferation (**Figure 4B**). MobMPP did not affect conventional (CD4^+^CD25^−^) T cell proliferation (**Figure 4C**). After four days of culture, naïve Treg cells were still Foxp3^+^ (**Figure 4D**). To further investigate how mobMPP could enhance naïve Treg proliferation, we first prevented cell-to-cell contact in transwell cultures. We observed that naive Treg proliferation, induced by mobMPP, was not totally abolished in transwell cultures (**Supplementary Data, Figure S2A**). However, neither naïve Treg cells nor mobMPP were able to produce IL-2, TNF-α, and IFN-γ, the main cytokines involved in T cell proliferation (**Supplementary Data, Figure S2B**). Taking into account a previously suggested mechanism in mice (9), and higher IL-1β production reported in CD34^+^ cells from PBSC relative to steady state bone marrow cells (10), we observed that 5% of freshly sorted mobMPP produce IL-1 β (**Supplementary Data, Figure S2C**). When neutralizing IL-1β by an IL-1β blocking antibody, naïve Treg survival and proliferation were reduced, although not fully abolished (**Supplementary Data, Figure S2D**).

## Discussion

Acute and chronic graft-versus-host diseases (GVHD) are major causes of morbidity and mortality after allo-HSCT. In mice, reduced frequency of Tregs contributes to both of these disorders (11). In humans, the incidence of natural Treg in peripheral blood (PB) is very low, amounting only to 1-2% (12), which is probably an overestimation because of contaminating CD25^+^ T-effector cells (13). This percentage is inversely correlated with the severity of acute (14) and chronic (14) GVHD. Among the strategies proposed to modulate and prevent GVHD, adoptive transfer of natural Tregs can reproducibly and efficiently prevent GVHD in murine models, but requires a high Treg dose (infusion of one Treg for one conventional donor T-cell) (15). Thus, in murine models, Treg expansion for adoptive transfer has been explored in a variety of approaches (16). In humans, co-cultures with diverse antigen-presenting cells have been investigated, such as donor-derived stimulated B cells, that expand the pool of alloreactive Tregs present in the blood (17, 18), as well as DCs (19) and artificial APCs (20). However, depending on the state of activation of Tregs, their proliferative capacity remains highly variable (21, 22) and the procedure is time-consuming. Furthermore, *ex vivo* expansion of natural Tregs (nTregs) must be anticipated far ahead of allo-HSCT. The safety and efficacy of *ex-vivo* expanded nTreg infusions have been attested (23, 24) and confirmed with a trend toward a lower incidence of acute grade II-IV GVHD. Even though, the technical obstacles associated with direct Treg adoptive transfer would make an alternative strategy leading to an enhancement of donor Tregs *in vivo* following allo-HSCT preferable. Mobilized MPP are interesting candidates for such an approach. They are readily available during HSCT, since the hematopoietic stem cells used are increasingly recovered from peripheral blood after G-CSF mobilization. Our evidence for the capacity of G-CSF-mobilized MPP to modulate autoimmunity (type 1 diabetes and EAE) through Foxp3^+^ Treg expansion and stabilization provides a further argument for their possible benefit during allo-HSCT. Starting from these findings, we explored whether their immunosuppressive functions applied also to allo-HSCT.

Our results show that mobMPP can selectively enhance survival and proliferation of activated Tregs *in vitro*, both in mice and humans. Their failure to promote proliferation of CD25^−^ cells may be considered an advantage over less selective options, such as IL-2 treatment (25), for which it is difficult to determine efficacious low doses, and foresee off-target effects, their short *in vivo* half-life being also problematic. Therefore, cellular therapy with mobilized MPP may provide a new method to expand *in vivo* alloantigen specific Tregs conferring protection against GVHD. Indeed, we observed that donor nTregs in mobMPP co-transferred recipients were rapidly expanded at allopriming sites and already exerted allospecific T-cell suppression at day-8 post-transplantation.

We observed that only mice given at least 0.35.10^6^ mobMPP had significantly improved survival. In fact, cohorts receiving 0.1.10^6^ mobMPP at the time of transplantation, did not significantly improve their survival (data not shown). The impact of higher mobMPP doses (>0.35.10^6^/mouse) on recipient outcomes was not studied. First, to avoid using excessive number of donor mice since one mobilized B6 mice was necessary to obtain 1.10^5^ mobMPP. Moreover, we assume that the dose of 3.5.10^5^ mobMPP represents a relevant ratio for coinjection with 2.10^6^ CD3^+^ T cells containing approximately 1.4 to 1.8.10^5^ natural Tregs in mice. Notably, the number of mobMPP required for protection in the allogeneic setting of GVHD is 10 to 30-fold higher than in autoimmune settings where as few as 10,000 and 25,000 mobMPP were sufficient to provide protection against spontaneous type 1 diabetes (7, 8) and Experimental Autoimmune Encephalomyelitis (9), respectively. This difference may reflect the multiple target tissues in GVHD where mobMPP may have to migrate to and control the allogeneic response, in contrast with the tissue specificity characterizing the autoimmune experimental settings.

Tregs from GVHD patients exhibit multiple defects, including instability of Foxp3 expression, impaired suppressive functions, decreased migratory capacity and increased apoptosis (26). Since we demonstrated the stability of MobMPP-expanded Tregs at the molecular level (9), we assume that *in vivo* expanded nTregs are less prone to differentiate into effector T cells during the cytokine storm post-HSCT (27, 28), a differentiation reported in inflammatory responses (29). In the same manuscript, we demonstrated that mobilized MPPs increase the transcription factor t-Bet in Tregs which enables them to suppress pathogenic Th1 cells and induces the expression of chemokine receptors, particularly CXCR3, which condition the migration of cells toward Th1 inflamed GVHD target organs (30). Finally, we demonstrated that Treg expansion by mobilized MPPs was partly mediated through IL-1β in mice. In the present study we identified a human mobMPP counterpart sharing most likely a similar mechanism to promote Treg expansion. IL-1β has been previously reported as a mediator involved in the induction of Foxp3^+^ Tregs in response to CD8α DCs exposed to GM-CSF (31). Moreover, these Tregs had a significantly higher ability to maintain Foxp3 expression when activated in the presence of IL-1β, which enhanced their capacity to suppress the effector T cell response *in vitro* and ongoing experimental autoimmune thyroiditis *in vivo* (32).

In humans the expression of inflammatory cytokine receptors IL-1R1 and TNFR2 has been described for being higher on resting mature Tregs than on naïve or memory T cells. While both receptors were upregulated upon activation through the T cell receptor (TCR), on all T cell subsets, IL-1R1 expression was maintained at significantly higher levels on activated Tregs than among other T cell subsets (33). Collectively, these results suggest that IL-1β-IL-1R signaling between mobMPP and Tregs could be a potent and safe mechanism to enhance natural Tregs *in vivo* in mice and should be explored in humans. It is noteworthy that the effect of IL-1β on GVHD depends critically on the timing of its intervention during allo-HSCT (34). Notably, in humans, IL-1β blockade during the period of initial T-cell activation had no impact on the cumulative incidence of acute GVHD (35).

Altogether, our results support the notion that mobilized MPPs could become useful as a novel cellular therapy to expand functional alloantigen specific Tregs *in vivo* that may lead to a considerable improvement of allo-HSCT safety by preventing GVHD.

## Data Availability Statement

The original contributions presented in the study are included in the article/**Supplementary Material**. Further inquiries can be directed to the corresponding author.

## Ethics Statement

The studies involving human participants were reviewed and approved by REG Allo NCTC02194868, CPP 24/04/2014, CCTIRS 18/06/2014, CNIL 17/04/2015. The patients/participants provided their written informed consent to participate in this study. The animal study was reviewed and approved by Paris Descartes University Ethical Committee and Ministry of Education and Research Ethical Committee (CEEA34.0AP.018.11).

## Author Contributions

MD’A and FZ designed the experiments, interpreted the data, and wrote the manuscript. MD’A, A-BN, VAA, AB, PG, MDC, and GF performed the experiments. MG-T provided technical support, and analyzed and interpreted the confocal microscopy. M-TR and OH provided the human HSC grafts. FZ, M-TR, and OH provided a critical review of the data and manuscript. All authors contributed to the article and approved the submitted version.

## Funding

This work was supported by the APHP (Assistance Publique des Hopitaux de Paris), and the “Institut National du Cancer”. FZ was supported by grants from Fondation ARC pour la Recherche contre le Cancer and from The Secular Society (TSS). VAA was recipient of a PhD fellowship from TSS.

## Conflict of Interest

The authors declare that the research was conducted in the absence of any commercial or financial relationships that could be construed as a potential conflict of interest.
