# Supplementary material for: Mobilized Multipotent Hematopoietic Progenitors Promote Expansion and Survival of Allogeneic Tregs and Protect Against Graft Versus Host Disease
Source: Front Immunol. 2021 Feb 12;11:607180. doi: 10.3389/fimmu.2020.607180 (PMC7907505; doi:10.3389/fimmu.2020.607180)
Supplement: Supplementary file 1 [file DataSheet_1.pdf]

D’Aveni et al.
Supplementary Figure 1

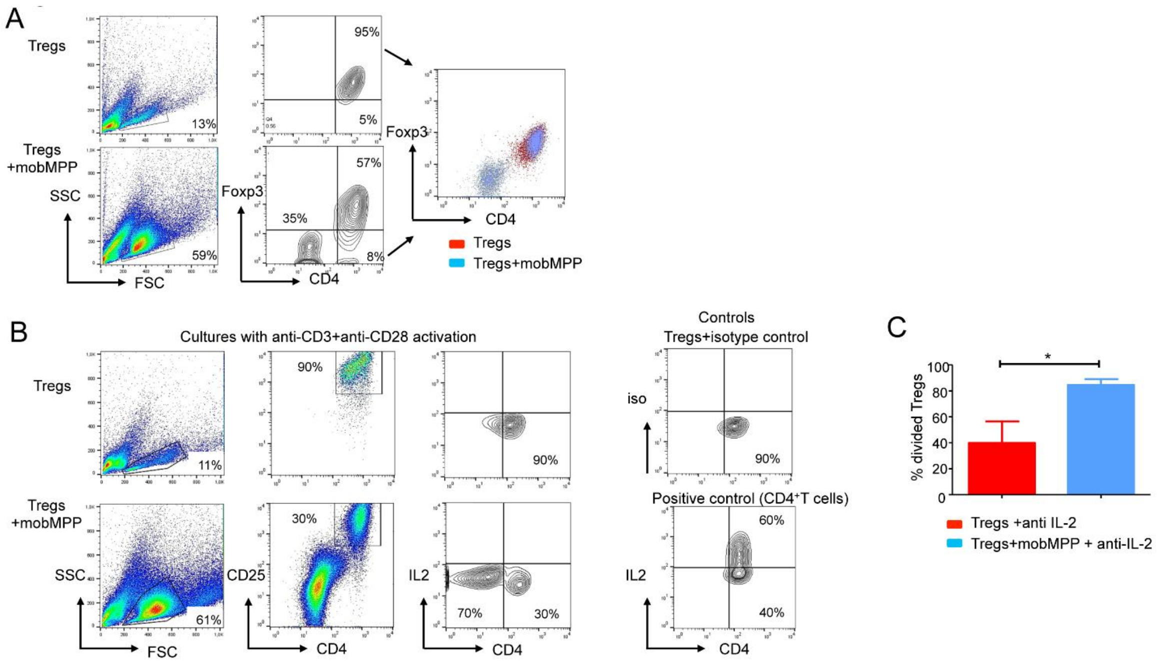

Figure S1. Natural Tregs enhanced by mobMPP maintain their phenotype and proliferate in an IL-2 independent mechanism.

A. B6 Treg cells activated by anti-CD3 and anti-CD28 were cultured alone or with mobMPP (1:1 ratio). After 4 days of culture, intra-nuclear Foxp3 was measured.

B. B6 Treg cells activated by anti-CD3 and anti-CD28 were cultured alone or with mobMPP (1:1 ratio). After 4 days of culture, cell surface molecules including CD4 and CD25 and intra-cytoplasmic IL-2 production were analyzed in Tregs and MobMPP. To assess intra-cellular IL-2 production, gates were based on isotype controls and positive control performed with B6 CD4+CD25- T cells activated by anti-CD3 and anti-CD28.

C. B6 Treg cells activated by anti-CD3 and anti-CD28 were cultured with IL-2 blocking antibody (red) or with mobMPP and IL-2 blocking antibody (blue), (n=3). The percentage of Tregs in proliferation (≥ division 1) is still significantly higher when Tregs are cultured with mobMPP even with blocking anti-IL-2 antibody (84.63 ± 2.53% vs. 39.87 ± 9.6%, *, p = 0.0107). Data from 3 independent experiments (n=3) were compared using Student’s unpaired t-test.
