# Supplementary material for: Mobilized Multipotent Hematopoietic Progenitors Promote Expansion and Survival of Allogeneic Tregs and Protect Against Graft Versus Host Disease
Source: Front Immunol. 2021 Feb 12;11:607180. doi: 10.3389/fimmu.2020.607180 (PMC7907505; doi:10.3389/fimmu.2020.607180)
Supplement: Supplementary file 2 [file DataSheet_2.pdf]

D'Aveni et al.
Supplementary Figure 2

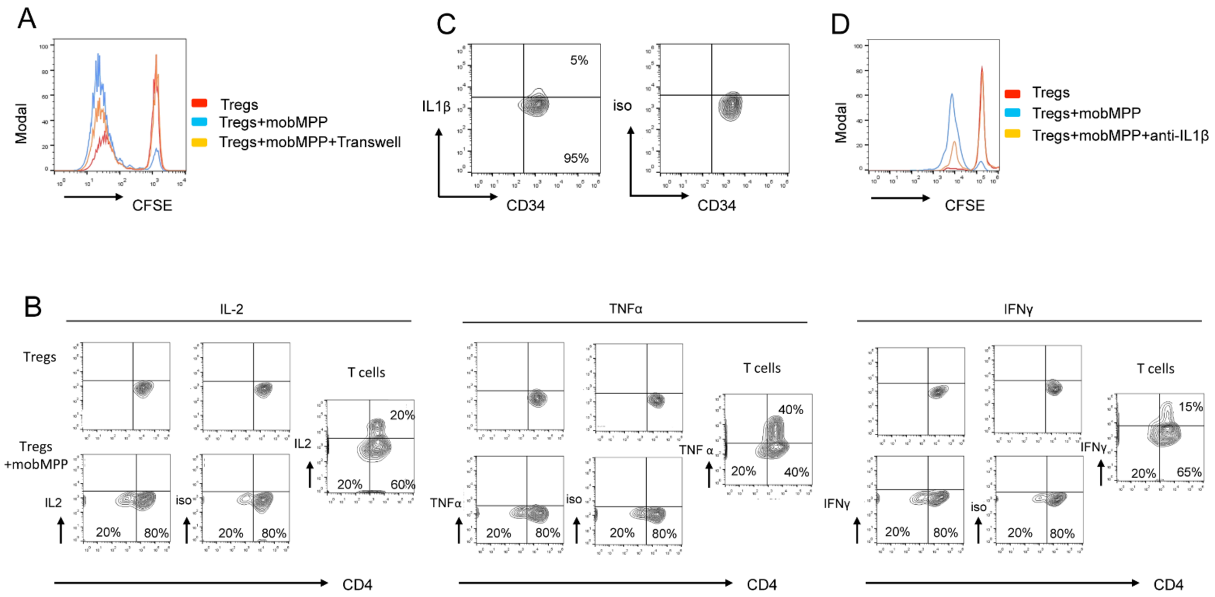

Figure S2. Human naïve Tregs enhanced by MobMPP in a mechanism including contact- and IL-1β dependency

(A) Anti-CD3 and -CD28 activated naïve Treg cell proliferation, assessed by CFSE dilution, after 4 days of culture alone (red) or with mobMPP either in contact (light blue) or with transwell device (orange).

(B) Naïve Treg cells activated by anti-CD3 and anti-CD28 were cultured alone or with mobMPP. After 4 days of culture, cell surface CD4 and intra-cytoplasmic IL-2, TNF-α and IFN-γ production were analyzed in Tregs and MobMPP. To assess intra-cellular cytokine production, gates were based on isotype controls and positive control performed with $CD4^{+}CD25^{-}$ T cells activated by anti-CD3 and antiCD28.

(C) Intracellular IL-1β expression in the mobMPP fraction (one representative experiment out of 4).

(D) Anti-CD3 and -CD28 activated naïve Treg cell proliferation after 4 days of culture alone (red), or with mobMPP in the presence (orange) or absence of 20 µg/ml anti-IL-1β (light blue), n=2.
